# Quantifying genetic variation for DUS descriptors in diverse finger millet germplasm evaluated under semi-arid Bundelkhand region of India

**DOI:** 10.3389/fpls.2026.1704652

**Published:** 2026-02-11

**Authors:** Dhanpati Keerthana, Vijay Kumar Yadav, Jitendra Kumar Tiwari, Rumana Khan, Subhash Chand, Dinesh Chandra Joshi, Shweta, Ram Vinod Kumar, Ravi Prakash Saini, Pankaj Kaushal, Anil Kumar

**Affiliations:** 1 Rani Lakshmi Bai Central Agricultural University, Jhansi, Uttar Pradesh, India; 2 ICAR-Indian Grassland and Fodder Research Institute, Jhansi, Uttar Pradesh, India; 3 Vivekananda Parvatiya Krishi Anusandhan Sansthan, Almora, Uttarakhand, India; 4 Chandra Shekhar Azad University of Agriculture and Technology, Kanpur, Uttar Pradesh, India

**Keywords:** cluster analysis, DUS traits, finger millet, genetic diversity, PCA

## Abstract

Finger millet (*Eleusine coracana* L.) is a nutritionally rich but underutilized crop found in semi-arid regions and drylands worldwide. To identify their genetically diverse and well-adapted genotypes to local conditions, a total of 162 germplasm lines, along with eight check varieties, were assessed during the rainy seasons of 2023 and 2024. The morphological characterization adhered to the Distinctiveness, Uniformity, and Stability (DUS) guidelines established by the PPV & FR Authority. The data collection concentrated on 14 qualitative traits and 13 quantitative traits. Significant variation was observed across all the traits. Specific traits such as earhead length, finger length, number of productive tillers, stover yield, and grain yield exhibited high heritability and significant genetic advance. The diversity index ranged from 0.162 for seed shape to 1.436 for ear shape. Grain yield demonstrated the most significant positive correlations with flag leaf width (0.503) and finger number (0.320). Cluster analysis classified the genotypes into six distinct clusters, with cluster 6 being highly diverse and possessing early maturing genotypes. PCA identified five principal components with eigenvalues greater than 1, collectively accounting for 70.1% of the total variation. The study identified genetically diverse and promising genotypes, establishing a solid foundation for targeted selection and breeding. The enhanced integration of molecular tools like genome-wide association studies may facilitate the precise selection of genotypes. The findings offer critical insights for selecting high-performing genotypes appropriate for cultivation and for improving breeding programs in this region.

## Introduction

Finger millet (*Eleusine coracana* L.) is a traditional and nutrient-dense cereal grain, extensively grown in Africa and Asia (Panwar et al., 2010). It is recognized for its exceptional resilience to harsh agroclimatic conditions and its capacity to thrive in diverse environments (Sood et al., 2022). It is a self-pollinating allotetraploid (2n = 4x = 36) crop characterized by finger-like panicles, which inspired its nomenclature (Upadhyaya et al., 2006). *E. coracana* subsp. coracana was domesticated from wild *E. coracana* subsp. africana around 5,000 years ago in the highlands between Ethiopia and Uganda (Neves et al., 2005). Finger millet is valued both as food crop in developing countries and as animal feed in developed nations. Often referred to as “poor man’s crop” due to its affordability (Ceasar et al., 2018; Wambi et al., 2020), it is nevertheless nutritionally superior to staple cereals like rice and wheat. Worldwide, it is regarded as the fourth most millet after sorghum (*Sorghum bicolor*), pearl millet (*Pennisetum glaucum*), and foxtail millet (*Setaria italica*) (Maharajan et al., 2019). Finger millet is rich in calcium, iron, magnesium, potassium, phosphorus, zinc, amino acids, and vitamins. Furthermore, absence of gluten in protein renders it a dependable option for those with gluten sensitivity (Saleh et al., 2013).

Finger millet is grown worldwide on around 2.1 million hectares, yielding over 3.7 million tonnes per year. India has the largest cropped area and production of finger millet, including over 1.0 million hectares (46% of the global area) and yielding 1.75 million tonnes annually. Ethiopia ranks second with 0.48 million hectares (22% of the world area) and a production value of 1.21 million tonnes (Gebreyohannes et al., 2021; FAOSTAT, 2020). In India, the productivity of finger millet has risen markedly from 649 kg/ha in 1950–1951 to 1,724 kg/ha in 2019–2020 (Hariprasanna, 2023). Millets, particularly finger millet, have historically made significant contributions to India’s nutritional security, sustainable agriculture, and rural livelihoods (Backiyalakshmi et al., 2024). Their inherent tolerance to environmental circumstances and nutritional advantages (Bandyopadhyay et al., 2023) make them essential crops in the global initiative to mitigate malnutrition and food insecurity. The promotion of millet cultivation is essential for achieving agricultural sustainability, improving dietary diversity, and fostering climate-resilient food systems (Mehta et al., 2024). The production of millets was fundamental to the sustainable food and fodder system in the Bundelkhand region; however, the area under millets has significantly diminished since the Green Revolution period. Consequently, the reinstatement and revitalization of finger millet production in these vulnerable regions may provide a climate-resilient approach to enhancing food security, nutrition, and adaptation to climate change.

Phenotypic evaluation is a reliable and practical first step in identifying promising and divergent lines. Assessing agro-morphological diversity enables the identification of genotypes that are adapted to local conditions and provides foundation for breeding programs (Kayastha et al., 2024). Diversity indices, genetic parameters, and multivariate analysis such as cluster analysis and principal component analysis (PCA) are widely used to characterize variability and parental selection (Likeng-Li-Ngue et al., 2025; Mondal et al., 2024). While phenotyping for DUS traits offers a clear picture of morphological variation and trait performance, integrating genotypic data could further strengthen the selection process by revealing the underlying genetic architecture. However, genotyping a large number of germplasm accessions can be time consuming and costly, making it challenging to apply at the initial screening stage. Therefore, the divergent genotypes identified through phenotypic assessment provide a valuable set of candidates for future genotyping, which can enhance precision in the selection.

The precise delineation and assessment of distinctness, uniformity, and stability (DUS) for traits such as plants, leaves, flowers, seeds, and phenological characteristics constitute the foundation for the protection of new plant varieties and genetic stocks by the International Union for the Protection of New Varieties of Plants (UPOV) and the Plant Variety Protection and Farmers Rights Authority in India. This research characterized and fully analyzed 27 DUS traits of 162 diverse finger millet genetic resources. The objective was to assess the phenotypic diversity and variation of DUS descriptors and to rank the examined phenotypic traits by multivariate analysis. The findings of this research will significantly aid in the conservation of finger millet genetic resources and the identification of prospective genetic resources with distinctive features. It further serves as a reference for the genetic enhancement of essential traits and offers a theoretical foundation for the future breeding of new cultivars.

## Materials and methods

### Experimental site and plant materials

The research was executed at the Indian Council of Agricultural Research - Indian Grassland and Fodder Research Institute located (coordinates 25^○^29′ N, 78^○^33′ E, and an elevation of 233 m.a.s.l.) in Jhansi, Uttar Pradesh, India. This study site is located in a semi-arid region characterized by unpredictable rainfall patterns and limited soil moisture availability.

A total of 162 finger millet genotypes along with eight promising check varieties namely: GPU 28, GPU 66, GPU 67, MR 1, MR 6, ML 365, PRM 2, and VR 929 were evaluated for DUS descriptors. Out of the 162 genotypes, 100 genotypes were obtained from the International Crops Research Institute for the Semi-Arid Tropics (ICRISAT) and 62 genotypes along with eight checks were procured from a national gene bank, ICAR-National Bureau of Plant Genetic Resources (NBPGR), New Delhi. The information on geographic and biological origin of the genotypes is furnished in **Supplementary Table 1**. The experiment was conducted using an Augmented Block Design as outlined by Federer and Ragava Rao (1975). The experimental design consists of six blocks, with each block accommodating 27 genotypes. Checks were included in each block, resulting in six replications, whereas each test genotype was represented only once. Each accession was planted in a two-row plot of 3 m in length, with a spacing of 30 cm between rows. Extra plants were removed within 3 weeks of planting to maintain a plant-to-plant spacing of 15 cm. In both the years, agronomic practices best suited for finger millet production were followed. The recommended N:P:K ratio, i.e., 40:20:0 kg/ha, respectively, was applied for raising the crop. Nitrogen was applied in two split doses. Half of the nitrogen was applied during land preparation, and the second half was applied after the first weeding, i.e., 30 days after planting. The total precipitation received during the crop growth period of 2023 was 18.75 mm, and that in 2024 was 25.86 mm.

### Evaluation of agro-morphological traits

Agro-morphological data were acquired on 27 distinct characteristics at relevant crop development stages in accordance with the DUS guidelines of the Protection of Plant Varieties and Farmers’ Rights Act (2019) for finger millet. During the two-to-four-leaf stage, observations were made on plant growth habit, pigmentation at the leaf juncture, leaf sheath pubescence, days to 50% flowering, and glume color during the flowering stage. Traits recorded during the dough stage included flag leaf length, flag leaf width, peduncle length, ear shape, finger branching, position of finger branching, multiple whorls, earhead length, finger length, finger width, and the number of fingers on the main ear. Post-harvest data were collected on the number of productive tillers, plant height, seed coverage by glumes, seed shattering, seed color, seed shape, seed surface, pericarp persistence, and 1,000 grain weight. Traits like plant growth habit, pigmentation at the leaf juncture, leaf sheath pubescence, glume color, ear shape, finger branching, multiple whorls, seed covering by glumes, seed shattering, seed color, seed shape, seed surface, and pericarp persistence were visually assessed at the plot level. Days to 50% flowering were counted as the number of days taken from sowing to the stage when ears have emerged from 50% of the main tiller of a genotype. Flag leaf length was measured from the ligule to the leaf tip, and width was measured at its widest point. Peduncle length was measured from the earhead base to the topmost node of the main tiller. Earhead length was taken from the base of the thumb finger to the tip of the longest finger. Finger length was measured from the base to the tip of the longest finger on the main ear, whereas finger width was measured at the widest point of the finger. Plant height was measured from the ground level to the tip of the earhead of the main tiller. For 1,000 grain weight, grains were counted manually for each accession and weighed in grams.

### Data analysis

#### Descriptive statistics

Frequency distribution and Shannon diversity index were performed using Microsoft Excel. ANOVA was performed by using a statistical procedure as outlined by Sharma (1998). The Shannon Weaver diversity index (1949) was calculated for all the traits using the given formula:


$$H^{\prime }=-\sum_{i=1}^{n}p_{i}\log_{e}p_{i}$$

where pi = n/N, n = number of individuals, and N = the total individuals.

#### Genetic parameters

The data from the two seasons of 2023 and 2024 were initially analyzed separately to assess year-wise variation. As the genotype × year interaction was non-significant, data were pooled across years for further analysis to assess genotype performance. Variance components and genetic parameters such as heritability in the broad sense and genetic advance as percent of the mean were computed from the pooled data by using “augmented RCBD” and “agricolae” packages in R software (4.5.0 version).

#### Multivariate analysis

The correlation analysis of the studied genotypes among the 13 quantitative traits was carried out using the “corrplot” function with the “pie” method. Clustering based on the ward method was performed using factoextra and NbClust packages. Principal component analysis (PCA) is a powerful tool in modern data analysis because this is a well-known multivariate statistical technique which is used to identify the minimum number of components, which can explain maximum variability out of the total variability (Anderson, 1972 and Morrison, 1978) and also to rank genotypes on the basis of PC scores. PCA was performed by using ggplot2 and factoextra packages in R.

## Results

### Morphological assessment

**Figure 1:**
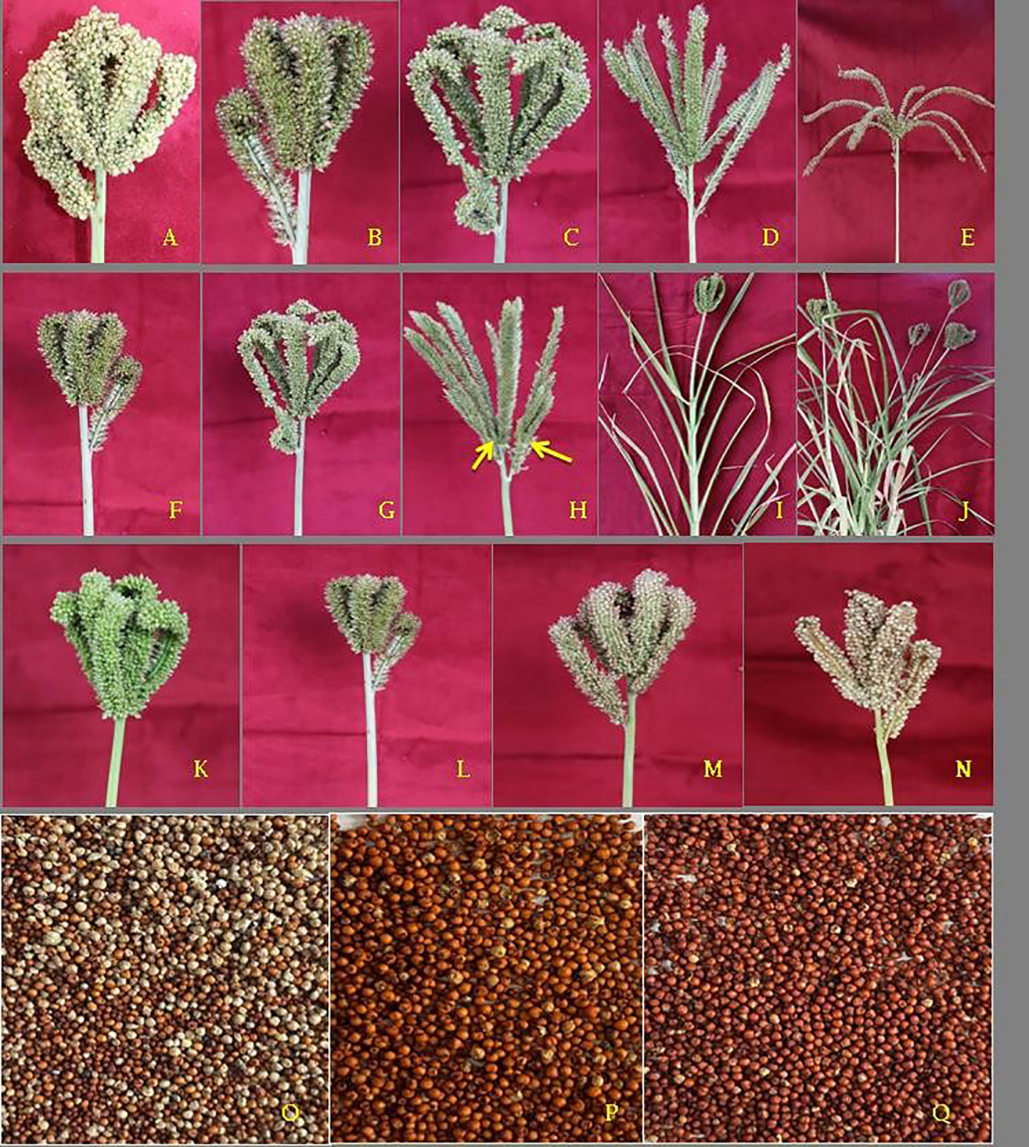
Various phenotypic characters recorded at different plant growth stages of finger millet. Ear shape—**(A)** fist (IC0073581); **(B)** compact (IC0087526); **(C)** semi-compact (IC0065595); **(D)** open (IC0049940); **(E)** droopy (IC0473384). Finger branching—**(F)** absent (IC0045878); **(G)** present: on thumb fingers (IC0049940); **(H)** all fingers (IC0204144). Plant pigmentation at leaf juncture— **(I)** absent (IC0065595); **(J)** present (IC0065632). Glume color—**(K)** green (IC0045878); **(L)** light purple (IC0204144); **(M)** dark purple (IC0475632); **(N)** white (IC0477899). Seed colors—**(O)** light brown (IC0065998); **(P)** dark brown (IC0050000); **(Q)** ragi brown (IC0283409).

**Figure 2:**
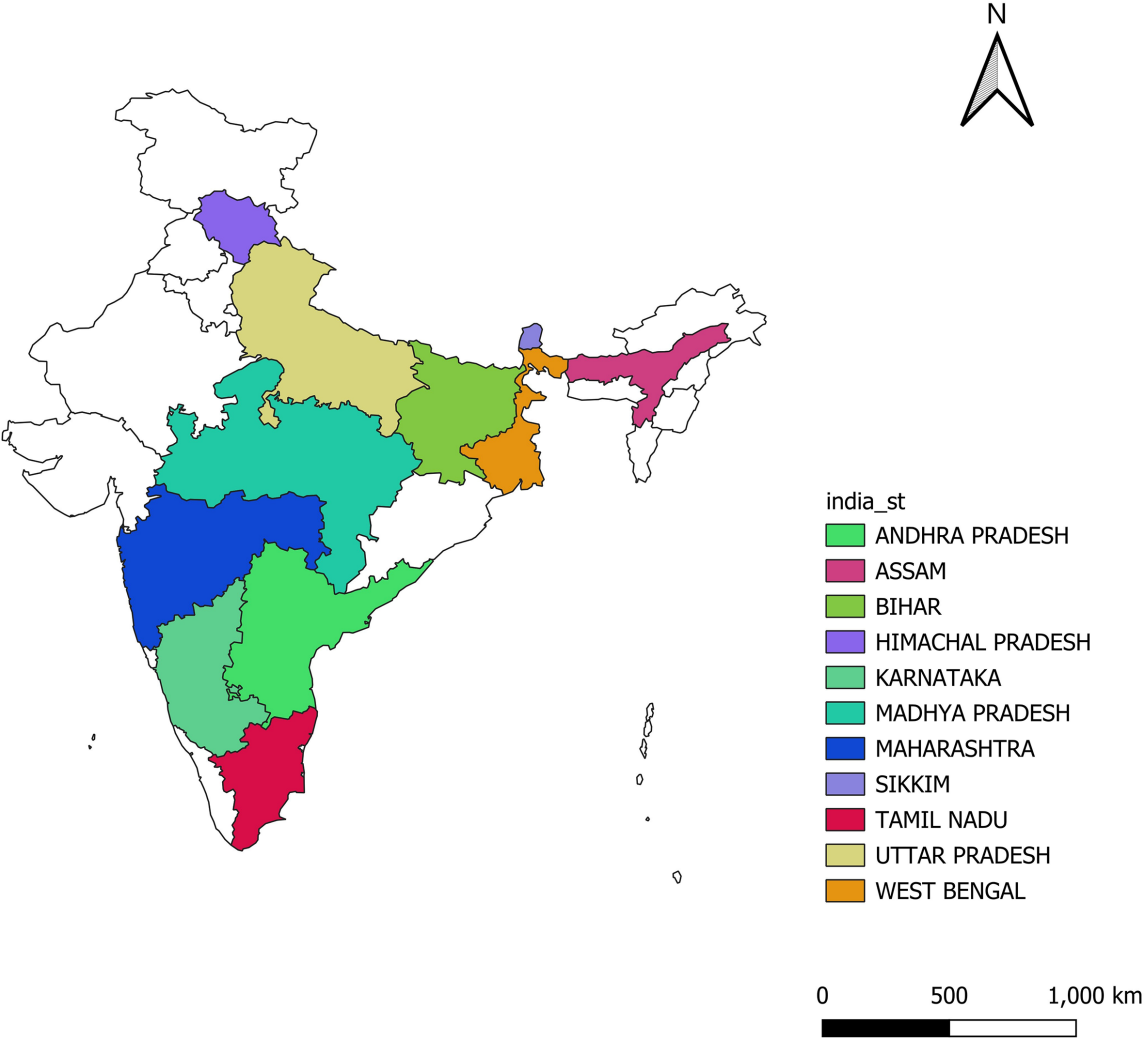
Genotypes collected from various states of India in the current investigation.

All the 162 genotypes along with eight checks were characterized as per DUS characters of finger millet to classify them into various groups and are presented in **Table 1** and **Figure 1**. Genotypes collected from various parts of India are presented in **Figure 2**. Plant growth habit is classified as 78.48% of erect (135), 18.6% of decumbent (32), and 1.74% of prostrate (03) type, indicating the predominance of erect growth habit across diverse millet germplasms. Pigmentation at the leaf juncture was present in 62 genotypes; the remaining 108 genotypes were non-pigmented. Leaf sheath pubescence was exhibited by 88 genotypes and absent in 82 genotypes providing insights into diversity for pubescence. Glume color was classified into three categories green, purple, and white. Majority of the genotypes (55.23%, 95) possessed a green-colored glume, followed by a purple-colored glume (43.02%, 74) and a single genotype with a white glume (0.58%, 1). Ear shape was compact and semi-compact in the 47 and 63 genotypes, respectively, followed by open type (32), fist (18), and then droopy (10). The finger branching trait was observed in 18 genotypes, whereas 152 genotypes were devoid of this. This variation provides important information on genetic diversity within the studied germplasm collection. Seed shattering, an important trait, was absent in 109 (63.37%) genotypes and present in 53 (30.81%) genotypes. A notable fraction of genotypes (97.93%, 165) exhibited a round seed shape; less fraction of genotypes were categorized into reniform (1.74%, 3) and ovoid (1.16%, 2). Majority of the studied genotypes fell under the persistent category (85.46%, 147) and the least majority under the non-persistent category (13.37%, 23). A total of 18 genotypes were of early maturing type (<55 days), and 152 genotypes were of medium maturing type (55–70 days). The phenotypic classification for flag leaf length categorized 46 genotypes into longest flag leaf lengths (30.1–45 cm) and 124 genotypes with moderate flag leaf widths (15–30 cm). Earhead lengths of 10 genotypes were short (4–6 cm); 63 were short to medium type (6.1–8 cm); 62 were of medium type (8.1–10 cm); 27 were long (10.1–12 cm); and 7 were of very long type (>12 cm). Among the studied genotypes for peduncle length, 114 were medium (20.1–30 cm) and 52 were long (30.1–40 cm). Longer and wider fingers contribute to higher grain yield by supporting more and better-developed spikelets. A total of 92 genotypes (53.49%) fell under the category of long finger length (>7 cm), 116 genotypes (67.44%) were of medium finger width (0.7-1.0 cm), and 45 genotypes (26.16%) were of wide finger width (>1.0 cm). Finger number on the main ear refers to the count of spike-like projections, commonly known as fingers, present on the earhead. It was categorized into low (6.4%, 11), medium (87.79, 151), and high (4.65%, 8) types. Most of the genotypes (93.6%, 161) studied possess a smaller number of productive tillers followed by moderate number (4.65%, 8) to high number (0.58%, 1). Plant height at maturity affects lodging resistance, harvestability, and overall biomass production. Most of the genotypes evaluated fell under the category of medium height (78.49%, 135); few were of short height (18.6%, 32) and more height (1.74%, 3). Higher weight often reflects well-filled, healthy grains. TGW was classified into three kinds, namely, low (5.23%, 9), medium (63.37%, 109), and high (30.23%, 52). Grain yield was found to be medium in majority of the studied genotypes (61.05%, 105).

**Table 1:** Characterization and frequency distribution of finger millet genotypes based on DUS traits.

S. no	Qualitative traits recorded	Descriptors scored		No. of accessions	RF (%)	Shannon index
1	Plant: growth habit (PGH)	1 -	Erect	135	78.48	0.573
		3 -	Decumbent	32	18.60	
		5 -	Prostate	3	1.74	
2	Plant: pigmentation at leaf juncture (PLJ)	1 -	Absent	108	62.79	0.660
		9 -	Present	62	36.04	
3	Leaf sheath pubescence (LSP)	1 -	Absent	82	47.67	0.696
		9 -	Present	88	51.16	
4	Glume: color (GC)	1 -	Green	95	55.23	0.879
		3 -	Light purple	9	5.23	
		5 -	Dark purple	65	37.79	
		7 -	White	1	0.58	
5	Ear: shape (ES)	1 -	Fist	18	10.46	1.436
		3 -	Compact	47	27.33	
		5 -	Semi-compact	63	36.62	
		7 -	Open	32	18.60	
		9 -	Droopy	10	5.81	
6	Finger: branching (FB)	1 -	Absent	152	88.37	0.345
		9 -	Present	18	10.46	
7	Finger: position of branching (FPB)	3 -	In thumb finger	15	8.72	0.306
		5 -	In all the fingers	155	90.11	
8	Finger: multiple whorl (FMW)	1 -	Absent	46	26.74	0.588
		9 -	Present	124	72.09	
9	Seed: shattering (SS)	1 -	Absent	109	63.37	0.652
		9 -	Present	53	30.81
10	Seed: covering by glumes (SCG)	2 -	Enclosed	21	12.21	0.381
		4 -	Intermediate	149	86.62	
		6 -	Exposed	0	0.00	
11	Seed: color (SC)	1 -	White	0	0.00	0.684
		3 -	Light brown	115	67.44	
		5 -	Dark brown	53	33.14	
		7 -	Ragi brown	2	4.07	
12	Seed: shape (SSP)	1 -	Round	165	95.93	0.162
		3 -	Reniform	3	1.74	
		5 -	Ovoid	2	1.16	
13	Seed: surface (ST)	3 -	Smooth	142	82.56	0.454
		7 -	Rough	28	16.28	
14	Pericarp: persistence after threshing (PPT)	1 -	Non-persistent	23	13.37	0.403
		9 -	Persistent	147	85.46	
15	Days to 50% flowering (DFF)	3 -	Early (<55 days)	18	10.47	0.345
		5 -	Medium (55–70 days)	152	88.38	
		7 -	Late (>70 days)	0	0.00	
16	Flag leaf length (FLL) (cm)	3 -	Short (<15)	0	0.00	0.589
		5 -	Medium (15.0-30.0)	124	72.09	
		7 -	Long (30.1-45)	46	26.74	
17	Flag leaf width (FLW) (cm)	3 -	Narrow (<1.0)	141	81.98	0.463
		5 -	Medium (1.0-2.0)	29	16.86	
		7 -	Wide (>2.0)	0	0.00	
18	Peduncle length (PL) (cm)	1 -	Short (10.0-20)	4	2.33	0.721
		3 -	Medium (20.1-30)	114	66.28	
		5 -	Long (30.1-40)	52	30.23	
19	Earhead length (EHL) (cm)	1 -	Very short (<4)	0	0.00	1.322
		3 -	Short (4.0-6.0)	10	5.81	
		4 -	Short to medium (6.1-8)	63	36.62	
		5 -	Medium (8.1 - 10)	62	36.05	
		7 -	Long (10.1 - 12)	27	15.69	
		9 -	Very long (>12)	7	4.07	
20	Finger length (FL) (cm)	3 -	Short (<5)	5	2.91	0.801
		5 -	Medium (5.0-7.0)	73	42.44	
		7 -	Long (>7)	92	53.49	
21	Finger width (FW) (cm)	3 -	Narrow (<0.7)	9	5.23	0.771
		5 -	Medium (0.7-1.0)	116	67.44	
		7 -	Wide (>1.0)	45	26.16	
22	Finger number on main ear (FN)	3 -	Low (<5)	11	6.40	0.432
		5 -	Medium (5.0-8.0)	151	87.79	
		7 -	High (>8)	8	4.65	
23	No. of productive tillers/plant (NPT)	3 -	Low (1-3)	161	93.60	0.234
		5 -	Medium (4-6)	8	4.65	
		7 -	High (>6)	1	0.58	
24	Plant height at maturity (PH) (cm)	3 -	Short (40-80)	32	18.60	0.574
		5 -	Medium (80-120)	135	78.49	
		7 -	High (>120)	3	1.74	
25	1,000 grain weight (TGW) (g)	3 -	Low (<2)	9	5.23	0. 805
		5 -	Medium (2-3)	109	63.37	
		7 -	High (>3)	52	30.23	
26	Stover yield (SY) (g/plant)		Low (up to 20g)	28	16.28	0.970
			Medium (21–40 g)	97	56.40	
			High (>40 g)	45	26.16	
27	Grain yield (GY) (g/plant)		Low (up to10g)	58	33.72	0.792
			Medium (10–20 g)	105	61.05	
			High (>20 g)	7	4.07	

PGH, plant: growth habit; PLJ, plant: pigmentation at leaf juncture; LSP, leaf sheath pubescence; GC, glume color; SS, seed: shattering; SCG, seed: covering by glumes; SC, seed: color; SSP, seed: shape; ST, seed: texture; PPT, pericarp: persistence after threshing; ES, ear shape; FB, finger: branching; FPB, finger: position of branching; FMW, finger: multiple whorl; DFF, days to 50% flowering; PH, plant height; PL, peduncle length; FLL, flag leaf blade: length; FLW, flag leaf blade: width; EHL, earhead length; FL, finger length; FW, finger width; FN, finger number; NPT, number of productive tillers; TGW, 1,000 grain weight; SY, stover yield; GY, grain yield

### Analysis of variance

The pooled analysis of variance (**Table 2**) revealed significant differences among genotypes, environments, and genotype × environment interactions for all 13 quantitative traits at the *p* = 0.01 significance level, indicating substantial genetic variability among the evaluated genotypes. The error mean sum of squares was minimal for most traits—namely, plant height, flag leaf width, earhead length, peduncle length, finger length, finger width, finger number on the main ear, number of productive tillers, thousand grain weight, stover yield, and grain yield, suggesting uniformity in experimental conditions.

**Table 2:** Pooled ANOVA for key traits in 170 finger millet genotypes evaluated across rainy seasons of 2023 and 2024.

Source		Genotype	Year	Year: block	Year: genotype
Df		169	1	10	169
Traits	Mean sum of squares (pooled)				
	DFF	11,086.22**	2.88	14.94	181.03
	PH	96,806.45**	170.87**	6,111.27**	132.24**
	FLL	6,179.35**	31.50**	8.37	24.03
	FLW	8.42**	1.28**	0.01	0.08**
	EHL	2,010.38**	11.45**	0.2	6.843**
	PL	7,107.2**	3.82**	0.27**	0.11**
	FL	1,525.48**	3.46**	2.18**	1.25**
	FW	9.14**	0.83**	0.1**	0.08**
	FN	4.17**	450.09**	2.75**	1.12**
	NPT	345.43**	0.31**	0.39**	0.49**
	TGW	149.18**	1.06**	0.49**	0.3**
	SY	51,332.3**	42.4**	1.29**	0.96**
	GY	33,611.74**	88.7**	5.89**	5.88

**p-0.01 level of significance

DFF, days to 50% flowering; PH, plant height; FLL, flag leaf blade: length; FLW, flag leaf blade: width; EHL, earhead length; PL, peduncle length; FL, finger length; FW, finger width; FN, finger number; NPT, number of productive tillers; TGW, 1,000 grain weight; SY, stover yield; GY, grain yield

### Genetic parameters and variance components

**Figure 3:**
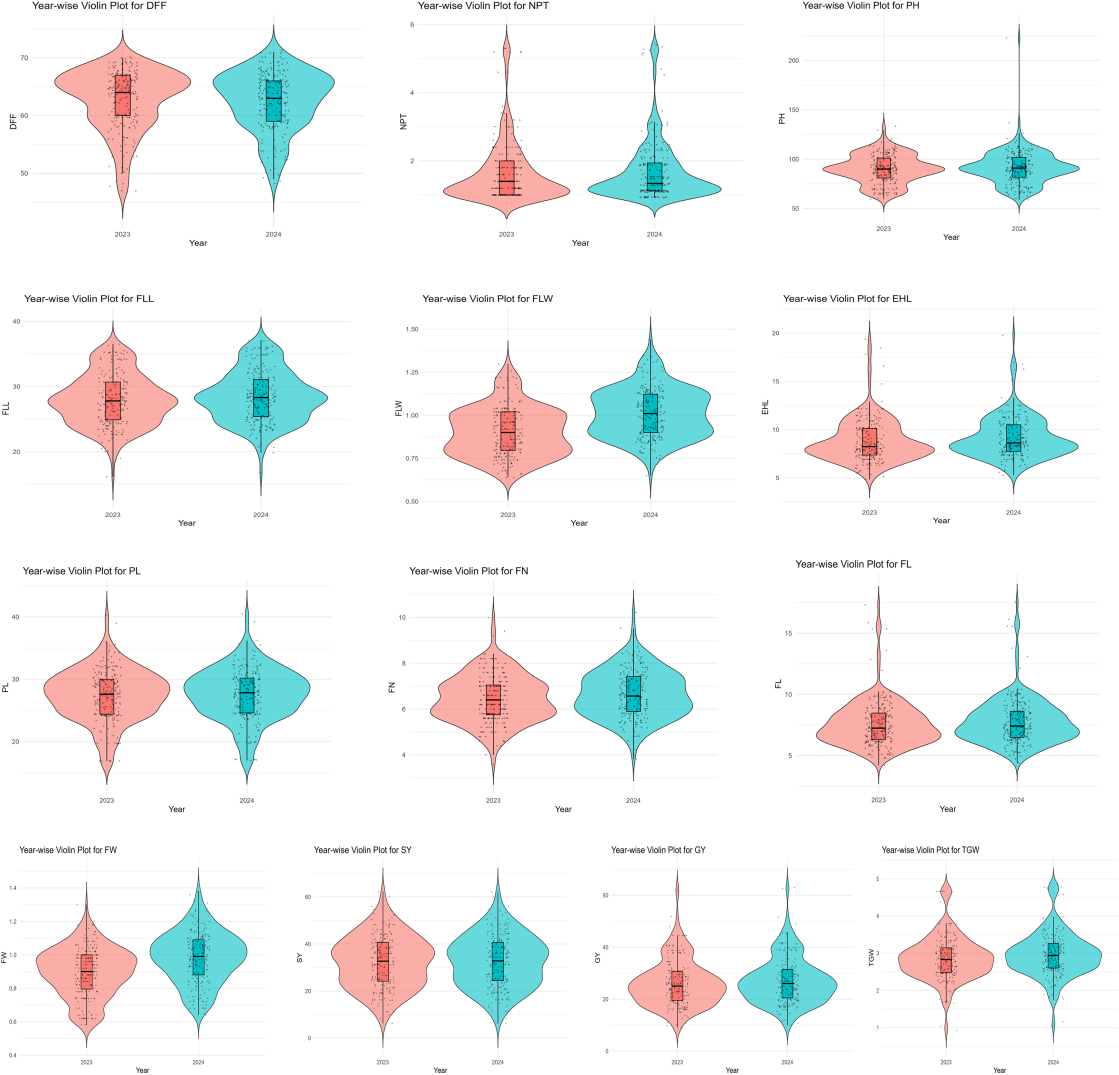
Violin plots illustrating the distribution of 13 quantitative traits in 170 finger millet accessions evaluated across two rainy seasons (2023 and 2024).

Trait-wise estimates of the mean, genotypic and phenotypic coefficients of variation (GCV and PCV), broad-sense heritability (H²), and genetic advance as a percentage of the mean (GAM) are shown in **Table 3**. Violin plots (**Figure 3**) were used to show the distribution, representing the density and spread of quantitative trait values in 170 finger millet genotypes over the years. For all the traits, GCV estimates were always less than the respective PCV estimates, reflecting the effect of environmental variation on the expression of the traits. Exceptionally high GCV and PCV estimates were found for earhead length (23.66%, 24.48%), finger length (24.97%, 25.56%), number of productive tillers (42.36%, 42.97%), stover yield (35.34%, 36.00%), and grain yield (31.77%, 32.65%), reflecting high genetic variability and great scope for selection. Moderate variability was observed for plant height, flag leaf characteristics, peduncle length, finger width, finger number, and 1,000 grain weight, with GCV and PCV of around 11% to 18%. High broad-sense heritability was observed for all the traits, and in combination with high GAM, this points toward the dominance of additive gene effects. The only exception was the days to 50% flowering, which had relatively lower heritability and genetic advance, indicating a higher impact of the environment and possible non-additive gene action.

**Table 3:** Pooled mean and genetic variability parameters estimated in studied finger millet accessions evaluated across two seasons.

Traits	Mean	Range		GCV (%)	PCV (%)	H^2^bs (%)	GAM (%)
		Min	Max				
DFF	62.22	48.26	71.08	7.82	9.01	75.38	14
PH	93.55	59.36	168.37	14.98	15.39	94.77	30.08
FLL	28.14	16.35	37	12.29	13.14	87.46	23.71
FLW	0.92	0.68	1.35	11.67	13.07	79.8	21.51
EHL	8.94	5.02	19.34	23.66	24.28	94.94	47.55
PL	28.31	16.75	40.11	12.19	12.8	90.59	23.93
FL	7.68	4.33	17.61	24.97	25.56	95.46	50.33
FW	0.97	0.61	1.31	11.85	12.69	87.09	22.81
FN	6.35	3.86	10.16	14.95	15.87	88.67	29.04
NPT	1.68	0.9	6.61	42.36	42.9	97.47	86.27
TGW	2.82	0.98	4.69	16.99	18.07	88.37	32.94
SY	31.92	7.03	62.98	35.34	36	96.33	71.55
GY	24.81	9.43	62.54	31.77	32.65	94.71	63.78

DFF, days to 50% flowering; PH, plant height; FLL, flag leaf blade: length; FLW, flag leaf blade: width; EHL, earhead length; PL, peduncle length; FL, finger length; FW, finger width; FN, finger number; NPT, number of productive tillers; TGW, 1,000 grain weight; SY, stover yield; GY, grain yield

### Shannon–Weaver Diversity (H′) index

**Figure 4:**
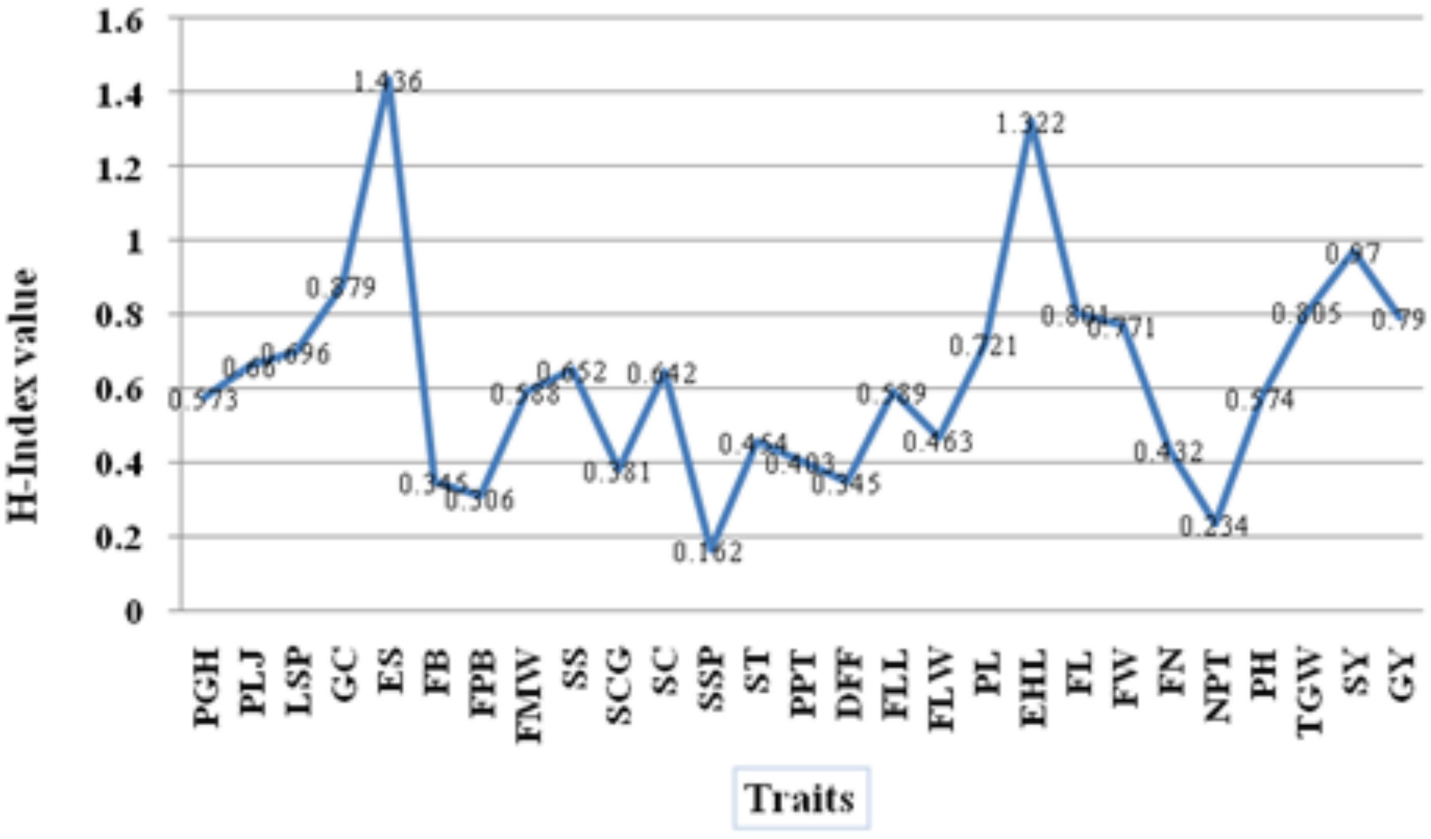
Shannon–Weaver diversity index (H′) among the studied traits in finger millet.

The Shannon–Weaver diversity index (H′) is a well-recognized measure of phenotypic diversity, as it considers both richness of traits and evenness of genotypes (Singh et al., 2024). In the present investigation, the H′ values for traits assessed are presented in **Table 1** and **Figure 4**. The H′ values for all traits ranged from 0.162 to 1.436, averaging 0.623, indicating a moderate level of total diversity in the finger millet genotypes under assessment. Among the many descriptors, ear shape showed the greatest diversity index (H′ = 1.436), followed by earhead length (1.322) and stover yield (0.970) expressing a high variation and scope for useful selection in these traits. Moderate variation was recorded for pigmentation at the leaf juncture (0.660), leaf sheath pubescence (0.696), and seed color (0.684). Conversely, seed shape had the least diversity index (0.162), followed by number of productive tillers (0.234) and finger branching position (0.345), implying less variability for these characteristics. Improvement in these traits requires incorporation of novel alleles or pre-breeding initiatives aimed at expanding the genetic base. The Shannon–Weaver index offers a valuable framework for pinpointing traits with significant variability that are suitable for selection to enhance germplasm.

### Pearson correlation

**Figure 5:**
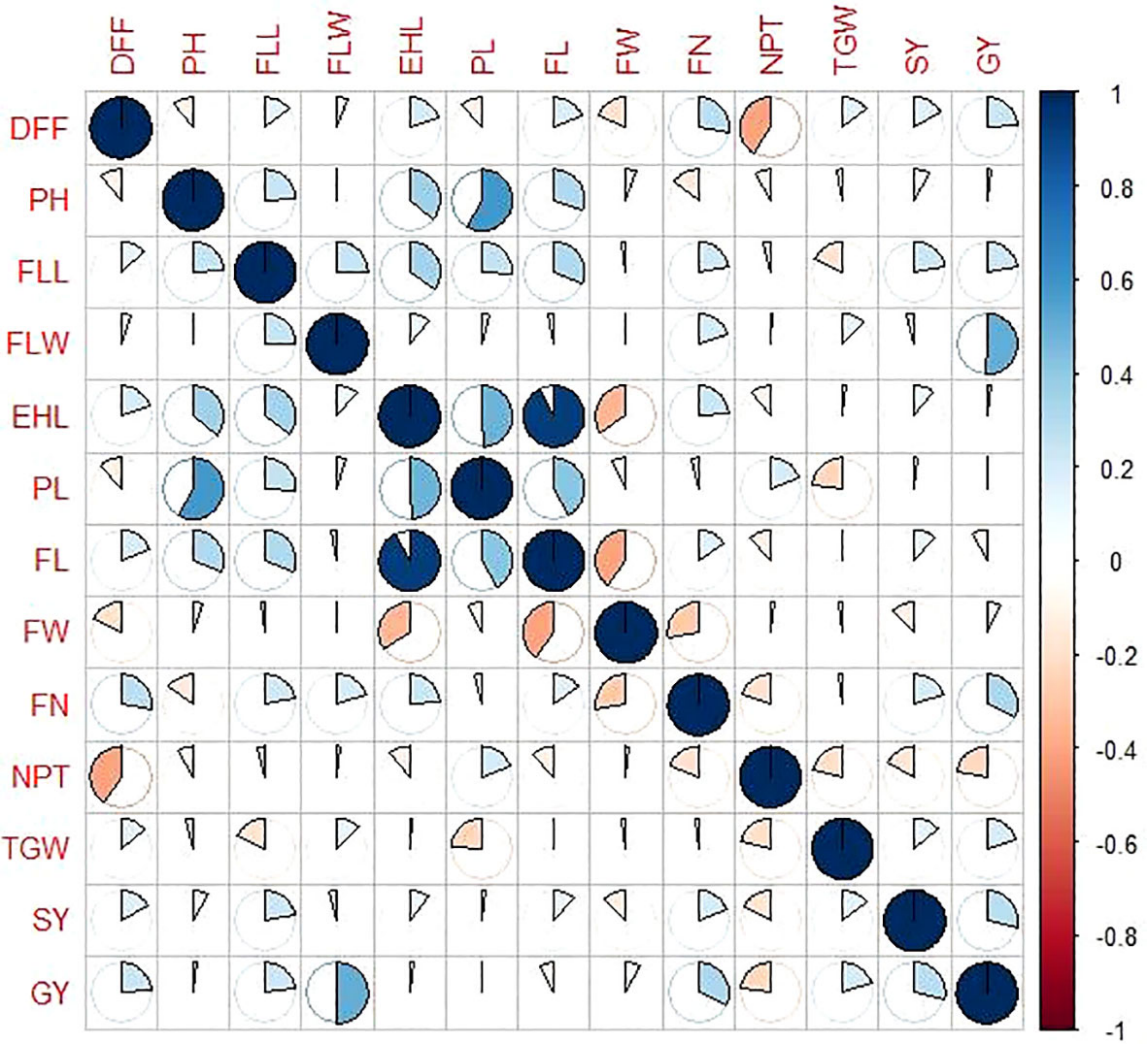
Correlogram for the various parameters studied in finger millet germplasm.

Correlation studies are important in assessing the directional relationship between any two variables. All traits showed a positive association with grain yield, except finger length and the number of productive tillers. A highly positive correlation was observed between earhead length and finger length (r = 0.923), peduncle length and plant height (r = 0.584), and flag leaf width and grain yield (r = 0.503). In contrast, days to 50% flowering exhibited a negative association with the number of productive tillers (r = –0.407), followed by finger length with finger width (r = –0.401), and earhead length with finger width (r = –0.344). Stover yield exhibited a negative association with the number of productive tillers. However, its positive correlation with other traits indicates that improvements in those parameters such as plant height and finger length can lead to increased stover output, making it a valuable component in dual-purpose breeding strategies. A correlogram plotted among the studied parameters is presented in **Figure 5** and **Table 4**.

**Table 4:** Trait associations between different quantitative traits studied in finger millet germplasm.

Traits	DFF	PH	FLL	FLW	EHL	PL	FL	FW	FN	NPT	TGW	SY	GY
DFF	1.000	−0.110	0.139	0.054	0.197	−0.117	0.188	−0.181	0.278*	−0.407*	0.142	0.166	0.238*
PH	−0.110	1.000	0.242	−0.005	0.358*	0.584*	0.307	0.052	−0.150	−0.086	−0.034	0.082	0.019
FLL	0.139	0.242	1.000	0.248	0.349	0.260	0.309	−0.024	0.217	−0.040	−0.180	0.222	0.223
FLW	0.054	−0.005	0.248	1.000	0.109	0.044	−0.033	−0.003	0.198	0.011	0.122	−0.042	0.503*
EHL	0.197	0.358*	0.349	0.109	1.000	0.487	0.923*	−0.344	0.231	−0.109	0.014	0.104	0.022
PL	−0.117	0.584*	0.260	0.044	0.487	1.000	0.419	−0.072	−0.037	0.197	−0.236	0.019	0.002
FL	0.188	0.307	0.309	−0.033	0.923*	0.419	1.000	−0.401	0.153	−0.118	0.001	0.113	−0.078
FW	−0.181	0.052	−0.024	−0.003	−0.344	−0.072	−0.401	1.000	−0.273	0.020	−0.023	−0.130	0.076
FN	0.278*	−0.150	0.217	0.198	0.231	−0.037	0.153	−0.273	1.000	−0.191	−0.018	0.191	0.320*
NPT	−0.407*	−0.086	−0.040	0.011	−0.109	0.197	−0.118	0.020	−0.191	1.000	−0.208	−0.173	−0.221
TGW	0.142	−0.034	−0.180	0.122	0.014	−0.236	0.001	−0.023	−0.018	−0.208	1.000	0.138	0.200
SY	0.166	0.082	0.222	−0.042	0.104	0.019	0.113	−0.130	0.191	−0.173	0.138	1.000	0.287*
GY	0.238*	0.019	0.223	0.503*	0.022	0.002	−0.078	0.076	0.320*	−0.221	0.200	0.287*	1.000

*p = 0.05 significance level

DFF, days to 50% flowering; PH, plant height; FLL, flag leaf blade: length; FLW, flag leaf blade: width; EHL, earhead length; PL, peduncle length; FL, finger length; FW, finger width; FN, finger number; NPT, number of productive tillers; TGW, 1,000 grain weight; SY, stover yield; GY, grain yield

### Multivariate analysis

Multivariate analysis encompasses a range of statistical techniques used to examine more than two variables simultaneously and helps to uncover relationships, patterns, and structural insights within complex datasets (Griffin et al., 2025). It helps in identifying key traits and supports efficient genotype classification. Techniques such as principal component analysis (PCA) and cluster analysis enable researchers to classify genotypes based on their similarities and identify genetically diverse, high-performing parents for use in breeding programs.

### Cluster analysis

**Figure 6:**
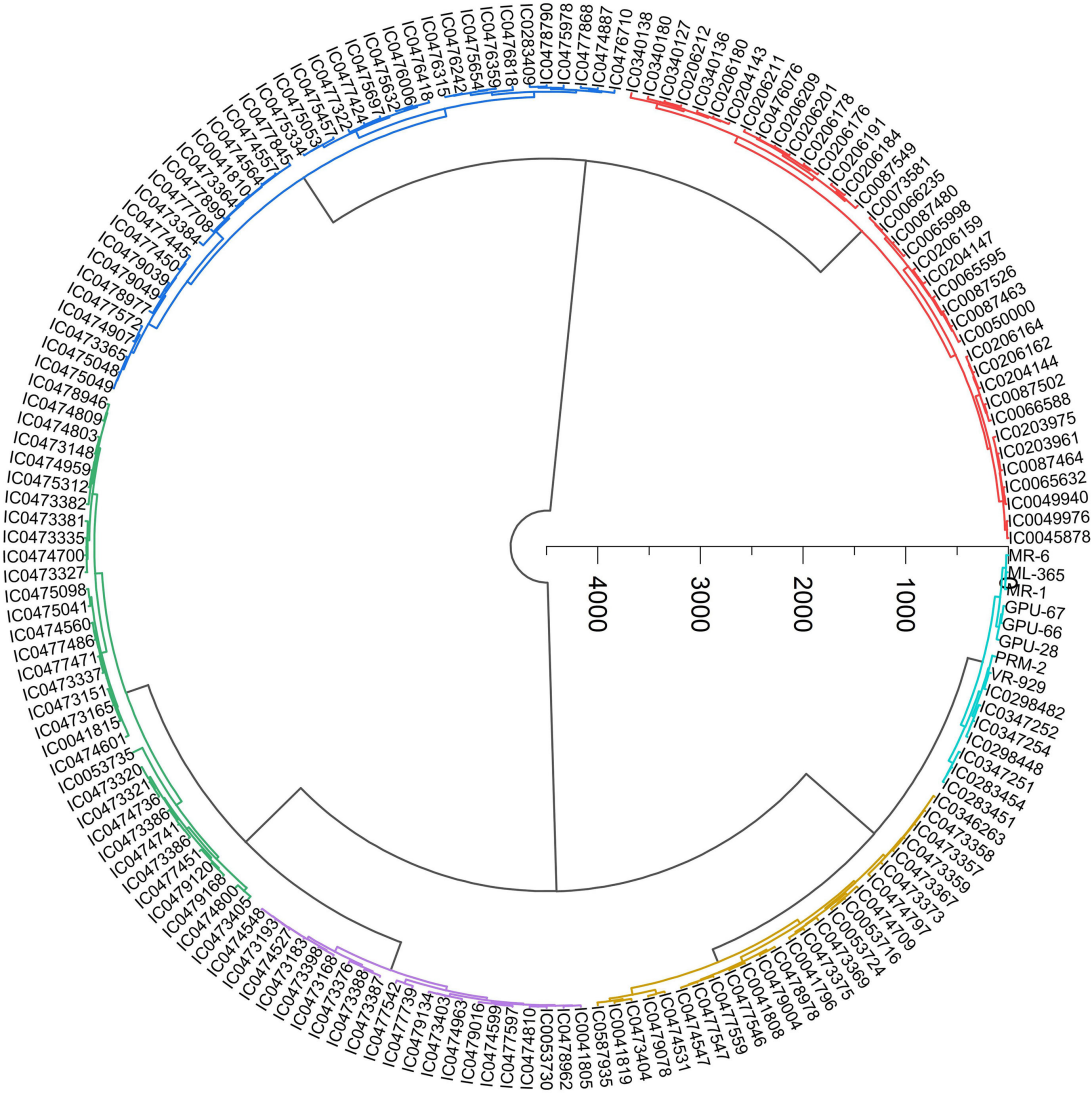
Dendrogram showing the distribution of 170 finger millet accessions for 13 quantitative traits across two seasons.

The 13 quantitative traits grouped the 162 finger millet genotypes and 8 checks into six distinct clusters, as illustrated in **Figure 6**. The six clusters were visually distinguished using different colors: red for cluster 1, dark blue for cluster 2, green for cluster 3, violet for cluster 4, yellow for cluster 5, and light blue for cluster 6. Clusters 1 and 2 were the largest, comprising 38 genotypes each, whereas cluster 6 was the smallest, with 15 genotypes. The intra- and inter-cluster D² values were estimated and are presented in **Table 5**. The highest inter-cluster distance (D² = 143.17) was observed between cluster 1 and cluster 6, followed by cluster 1 and cluster 5 (D² = 123.14) and cluster 2 and cluster 6 (D² = 105.31). In contrast, the shortest inter-cluster distance was recorded between cluster 6 and cluster 5 (D² = 29.22), followed by cluster 4 and cluster 5 (D² = 31.84), suggesting that the genotypes in these groups share the highest similarity. Cluster 6 exhibited the greatest variability within itself, as indicated by the highest intra-cluster distance (83.94), followed by cluster 1 (73.43) and cluster 5 (68.01). Among these, cluster 1 exhibited superior mean performance for stover yield and flag leaf length, indicating the presence of desirable genotypes for yield enhancement. Clusters 2 and 3 showed comparable values for traits such as days to 50% flowering and flag leaf dimensions, along with moderate grain yield. Early flowering genotypes were found in cluster 6, and high-yielding genotypes were found in cluster 5. Cluster means are presented in **Table 6**.

**Table 5:** Average inter and intra (diagonal in bold) cluster distance (Euclidean) in studied finger millet genotypes.

	Cluster1	Cluster2	Cluster3	Cluster4	Cluster5	Cluster6
Cluster1	**73.43**					
Cluster2	39.11	**53.60**				
Cluster3	79.49	43.31	**51.90**			
Cluster4	97.13	59.23	32.57	**55.30**		
Cluster5	123.14	85.75	50.44	31.84	**68.01**	
Cluster6	143.17	105.31	74.17	47.27	29.22	**83.94**
Cluster Size	38	38	33	21	25	15

Bold values refers to Intra cluster distances.

**Table 6:** Cluster means of 170 finger millet genotypes.

	1	2	3	4	5	6
DFF	64.92	60.20	60.74	64.00	63.90	58.47
PH	89.25	90.86	110.99	86.30	97.14	79.12
FLL	30.65	25.97	28.77	27.44	27.95	27.26
FLW	0.84	0.82	0.94	0.96	1.03	1.06
EHL	9.04	8.79	9.44	9.24	8.83	8.13
PL	27.64	27.96	30.24	27.55	30.05	25.19
FL	7.97	8.08	7.97	7.67	7.05	6.69
FW	0.96	0.97	0.98	0.99	1.02	0.89
FN	6.72	6.29	5.95	6.05	6.43	6.65
NPT	1.63	1.79	1.56	1.41	1.66	2.16
TGW	2.63	2.88	2.88	2.88	2.78	3.04
SY	37.48	33.39	27.81	24.60	36.05	27.82
GY	24.48	20.33	23.74	24.35	32.12	27.61

DFF, days to 50% flowering; PH, plant height; FLL, flag leaf blade: length; FLW, flag leaf blade: width; EHL, earhead length; PL, peduncle length; FL, finger length; FW, finger width; FN, finger number; NPT, number of productive tillers; TGW, 1,000 grain weight; SY, stover yield; GY, grain yield.

Cluster 2, despite exhibiting favorable performance for several yield-contributing traits, recorded the lowest grain yield, indicating that enhancement of a few individual traits alone does not necessarily translate into higher productivity. This reinforces the polygenic and complex nature of grain yield, which depends on the coordinated expression of multiple traits rather than isolated morphological parameters. The pattern of Mahalanobis D² statistics revealed that intra-cluster genetic distances were generally smaller than inter-cluster distances, confirming substantial genetic divergence among clusters. Interestingly, the genetic relationship between cluster 1 and cluster 2 was closer than the diversity observed within certain individual clusters, suggesting partial overlap in their genetic backgrounds. Such anomalies highlight the importance of considering both intra- and inter-cluster genetic divergence when selecting parents, ensuring that combinations chosen for hybridization truly maximize diversity and facilitate the development of heterotic and high-yielding segregants.

Cluster 2, despite performing well in several traits, had the lowest grain yield, demonstrating that yield depends on the synergistic contribution of multiple traits rather than on any single morphological parameter. Furthermore, the intra-cluster genetic distances were consistently lower than the inter-cluster distances. Interestingly, the genetic distance between cluster 1 and cluster 2 was smaller than the intra-cluster variation observed within certain individual clusters, denoting greater diversity within those clusters compared with the divergence between them. This anomaly implies that genotypes from these two clusters are genetically closer to each other than the genotypes within some single clusters, such as clusters 3 and 4. Henceforth, combining both intra and inter-cluster information before selecting parental lines may reflect true genetic divergence for development of superior hybrids in further breeding efforts.

### Principal component analysis

Principal component analysis (PCA) was employed to reduce data dimensionality while retaining maximum variability across the 13 quantitative traits evaluated in 162 finger millet genotypes along with 8 checks. This method transforms correlated variables into a smaller set of uncorrelated principal components (PCs), which facilitate the identification of patterns and major trait contributors to genetic diversity. The analysis yielded 13 PCs with eigenvalues ranging from 0.057 to 3.042 (**Table 7**). Five components with eigenvalues >1 cumulatively explained 70.1% of the total variation. PC1 contributed the highest proportion of variation (23.4%), followed by PC2 (17.2%) and PC3 (11.9%), cumulatively accounting for 52.5% of the total variance. The later components contributed minimal variation.

**Table 7:** Principal components (PCs) for 13 qualitative traits studied among the genotypes of finger millet characterized during rainy seasons of 2023 and 2024.

Traits	PC1	PC2	PC3	PC4	PC5	PC6	PC7	PC8	PC9	PC10	PC11	PC12	PC13
DFF	−0.195	0.368	0.237	−0.070	−0.138	0.368	−0.128	−0.663	−0.265	−0.209	−0.176	0.104	−0.015
PH	−0.272	−0.287	−0.250	−0.452	−0.028	0.015	0.326	−0.033	0.220	−0.340	−0.124	0.537	−0.031
FLL	−0.325	0.024	−0.315	0.141	−0.339	0.092	−0.569	0.135	0.284	−0.388	0.231	−0.153	0.019
FLW	−0.123	0.244	−0.481	0.247	0.454	0.095	−0.056	−0.090	0.288	0.177	−0.532	−0.032	−0.091
EHL	−0.505	−0.129	0.140	0.012	0.200	0.076	−0.139	0.165	−0.187	0.240	−0.043	0.113	0.713
PL	−0.323	−0.375	−0.241	−0.052	0.008	−0.045	0.316	−0.265	−0.248	−0.070	0.000	−0.673	−0.057
FL	−0.477	−0.158	0.257	−0.007	0.152	0.045	−0.209	0.153	−0.118	0.286	0.076	0.138	−0.685
FW	0.242	−0.040	−0.416	−0.338	−0.171	0.317	−0.237	0.295	−0.557	0.134	−0.218	0.027	−0.057
FN	−0.224	0.331	0.044	0.410	−0.148	−0.009	0.421	0.458	−0.307	−0.360	−0.173	0.049	−0.074
NPT	0.133	−0.341	−0.180	0.435	0.162	−0.401	−0.247	−0.265	−0.398	−0.220	0.007	0.346	−0.014
TGW	0.005	0.288	0.101	−0.435	0.567	−0.291	−0.204	0.146	−0.141	−0.432	0.060	−0.194	−0.015
SY	−0.182	0.229	−0.017	−0.214	−0.439	−0.696	−0.103	−0.064	−0.014	0.246	−0.340	−0.019	0.008
GY	−0.144	0.421	−0.435	−0.033	0.065	−0.082	0.202	−0.139	−0.154	0.268	0.643	0.178	0.002
Standard deviation	1.744	1.495	1.246	1.112	1.021	0.961	0.825	0.771	0.741	0.684	0.581	0.531	0.240
Proportion of Variance	0.234	0.175	0.119	0.095	0.080	0.071	0.052	0.046	0.042	0.036	0.026	0.022	0.004
Cumulative Proportion	0.234	0.406	0.525	0.620	0.701	0.772	0.824	0.870	0.912	0.948	0.974	0.996	1.000
Eigenvalue	3.042	2.234	1.552	1.237	1.042	0.923	0.680	0.595	0.549	0.467	0.338	0.282	0.057

DFF, days to 50% flowering; PH, plant height; FLL, flag leaf blade: length; FLW, flag leaf blade: width; EHL, earhead length; PL, peduncle length; FL, finger length; FW, finger width; FN, finger number; NPT, number of productive tillers; TGW, 1,000 grain weight; SY, stover yield; GY, grain yield

Trait loadings revealed that PC1 was primarily determined by finger width (0.242), number of productive tillers (0.133), and 1,000-grain weight (0.005), with a negative relationship for days to 50% flowering (–0.195). PC2 was notably related with grain yield (0.421), days to 50% flowering (0.368), finger number (0.331), 1,000-grain weight (0.288), and flag leaf attributes, reflecting its importance for yield and phenology-related traits. PC3 was dominated by finger length (0.257), earhead length (0.140), and 1,000-grain weight (0.101). PC4 was controlled by number of productive tillers (0.435), finger number (0.410), and flag leaf width (0.247), whereas PC5 was controlled by 1,000-grain weight (0.567) and flag leaf width (0.454), with earhead and finger length characters showing moderate contributions. Interestingly, days to 50% flowering always had a negative loading in PC1, PC4, and PC5, suggesting a possible inverse correlation with yield-related characteristics. Genotypes that have higher values for these components are expected to have desirable characteristics like finger width, more tillers, higher leaf area, and better grain filling—traits essential for developing high yielding and earliness.

**Figure 7:**
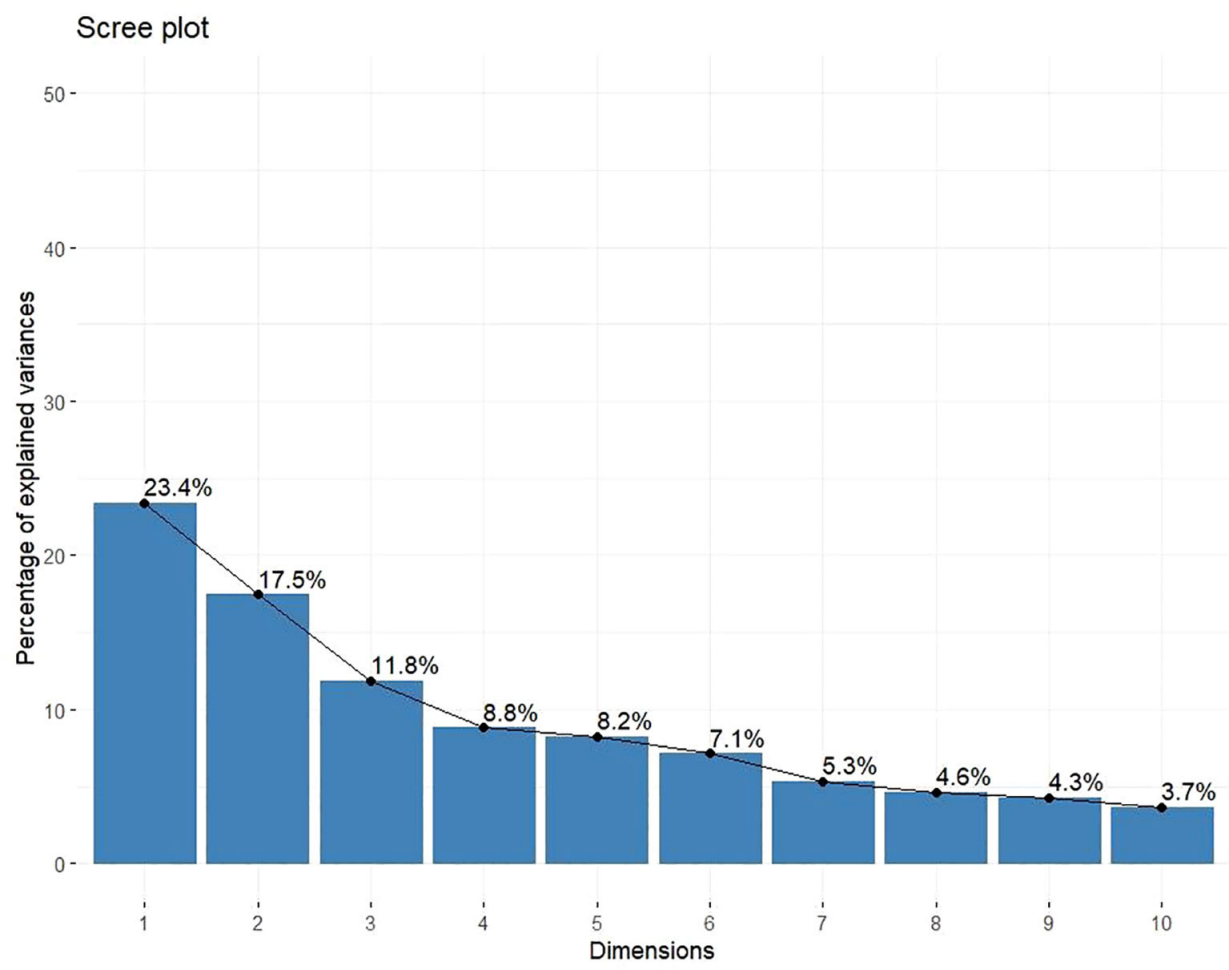
Scree plot showing explained variation of the first 10 principal components.

A scree plot (**Figure 7**) presented the variance explained by the first 10 PCs, whereas the biplot of PC1 *vs.* PC2 showed the trait associations and genotype distribution. Strong positive correlations were shown by acute angles between vectors in the biplot, whereas obtuse angles revealed negative or weak associations.

## Discussion

Agro-morphological characterization is a crucial method in plant breeding for evaluating genetic variability and comprehending trait connections in finger millet (*Eleusine coracana*). The current study revealed significant phenotypic diversity in both qualitative and quantitative traits (**Table 2**), especially in ear shape, earhead length, finger length, stover yield, 1,000 grain weight, and glume color, as evidenced by their high Shannon diversity index values. These characteristics also had a crucial role in principle component dimensions, highlighting their importance for genotype distinction. The observed morphological diversity aligns with previous research findings in finger millet. Choudhary et al. (2025) identified significant phenotypic variation among DUS descriptors in 66 finger millet genotypes, whereas Kumar et al. (2019) observed comparable diversity through 16 qualitative traits. The results indicate that phenotypic differentiation in finger millet may be attributed to historical landrace evolution, natural cross-pollination, or interspecific introgression. Moreover, the morphological assessment using DUS remains a rapid and economical approach for the preliminary evaluation and screening of germplasm in breeding programs.

ANOVA using an augmented block design demonstrated a high level of genetic variability by revealing significant genotype differences for each of the 13 quantitative characteristics. In their study of 88 genotypes, Andualem and Tadesse (2011) also observed comparable results, demonstrating the usefulness of such designs for preliminary screening. The resulting significant variance provides an excellent platform for selection of elite genotypes in breeding programs. Superior performing genotypes for various quantitative traits are provided in the **Table 8**.

**Table 8:** Genotypes exhibiting higher diversity for the important quantitative traits.

S. no.	Trait	Genotypes
1.	Days to 50% flowering	IC0476710, IC0476818, IC0476315, IC0475457, IC0474887, IC0347251, IC0347252, IC0347254
2.	Plant height	IC0065595, IC0066588, IC0087526, IC0204147, IC0473148, IC0473165, IC0053735, IC0473151
3	Flag leaf length	IC0066588, IC0073581, IC0087549, IC0474809, IC0473335
4	Flag leaf width	IC0065632, IC0087463, IC0087526,IC0087549, IC0203961, IC0476006, IC0204143
5	Peduncle length	IC0087526, IC0206159, IC0206162, IC0340138, IC0476006, IC0041810, IC0473364, IC0477597
6	Finger number on main ear	IC0049940, IC0066235, IC0066588, IC0087480, IC0204147, IC0479039
7	Number of productive tillers	IC0476006, IC0479134, IC0347251, IC0475632, IC0474887, IC0478790, IC0087463, IC0087526
8	Thousand grain weight	IC0049976, IC0475312, IC0475049, IC0477868, IC0476359, IC0476315, IC0206184
9	Stover yield	IC0065998, IC0049976, IC0065595, IC0073581, IC0087480, IC0475978, IC0476710, IC0087549
10	Grain yield	IC0204147, IC0479078, IC0587935, IC0474531, IC0041819, IC0473381, IC0477899

High genotypic and phenotypic coefficients of variation (GCV and PCV) for traits like earhead length, finger length, number of productive tillers, stover yield, and grain yield indicated the presence of valuable genetic diversity. These results are consistent with those of Nagaraja et al. (2023), who found similar variations in grain yield, finger length, and number of productive tillers. Plant height, flag leaf size, peduncle length, finger number, and 1,000-grain weight varied moderately, which is consistent with findings by Udamala et al. (2022). The majority of the attributes had high heritability and strong genetic advance as a percentage of the mean, indicating additive gene action and a good scope for improvement by selection.

A reliable indicator of phenotypic variability is the Shannon diversity index (H′). High H′ values for earhead length and ear shape, which is directly related to grain yield, signifies their overall importance in contributing to crop diversity. High phenotypic diversity makes it easier to distinguish genotypes and provide a foundation for future crop improvement programs. Singh et al. (2024) noted similar patterns, with a high degree of diversity in the shape of the earhead and a lower degree of variability for seed shattering. Ghimire et al. (2020) confirmed the validity of this diversity index by observing moderate to high H′ values in most of the traits. The high estimates of diversity index observed in the present study might be attributed to the diverse geographic and biological origin of the genotypes.

Grain yield and other characteristics, such as flag leaf width, finger number, stover yield, and days to 50% flowering, showed highly significant positive correlations. The direct perception of sunlight and crop photosynthetic activity increases with flag leaf width, which eventually leads to enhanced grain yield. The findings are consistent with those of John (2006), Chavan et al. (2020), and Jadhav et al. (2015), who all reported positive associations with grain yield. Under ideal circumstances, high stover yields typically coincide with higher grain yields because both are products of total biomass accumulation and photosynthetic efficiency. These findings are consistent with those of Dhami et al. (2018), who found that flag leaf length and grain yield were positively correlated with stover yield. Conversely, number of productive tillers showed negative associations with days to 50% flowering, suggesting a potential trade-off between early maturity and yield components. Eric et al. (2016) also observed similar trends and emphasized the importance of comprehending trait interactions in selecting genotypes for breeding programs.

Cluster analysis uses genetic similarity to group the genotypes (Semman et al., 2025). Using Ward’s method, 170 genotypes that were characterized for 13 quantitative traits were divided into six distinct clusters in the current study, demonstrating significant genetic divergence of germplasm. Cluster 1 exhibits germplasm with larger flag leaf length, finger number, stover yield, and late flowering. Cluster 2 is defined by germplasm exhibiting the higher finger length. Cluster 3 comprises germplasm exhibiting the higher plant height, earhead length, and peduncle length. Cluster 4 lacks germplasm exhibiting the highest values for any morphological trait. Cluster 5 is defined by germplasm exhibiting the highest finger width and grain yield. Cluster 6 is defined by germplasm exhibiting early flowering, maximum flag leaf width, number of productive tillers, and thousand grain weight. The highest genetic divergence occurred between cluster 1 and cluster 6, as evidenced by the maximum inter-cluster distance of 143.17, followed by the distance between cluster 5 and cluster 6 at 139.22. The high divergence primarily results from contrasting trait profiles. Cluster 6 includes early-flowering, short-statured genotypes characterized by high number of productive tillers and increased test weight. In contrast, cluster 1 comprises medium-maturing, taller plants that exhibit higher biomass and reduced tillering ability. Consequently, cluster 6 exhibits the greatest genetic divergence, demonstrating the highest distance from cluster 1, cluster 5, and cluster 3. The high genetic divergence between the clusters might be attributed to grouping of genotypes from different geographical and biological origins in different clusters. Significant distances between the clusters increase the likelihood of significant heterotic responses during hybridization by aiding in the selection of diverse parents. These results are consistent with previous findings by Sharma et al. (2022), who reported seven clusters in 31 genotypes. Additionally, in both larger and smaller data sets, Patil et al. (2017) and Anuradha et al. (2017) revealed unequivocal patterns of clustering, confirming the effectiveness of multivariate methods in identifying genetically diverse parents for hybridization.

**Figure 8:**
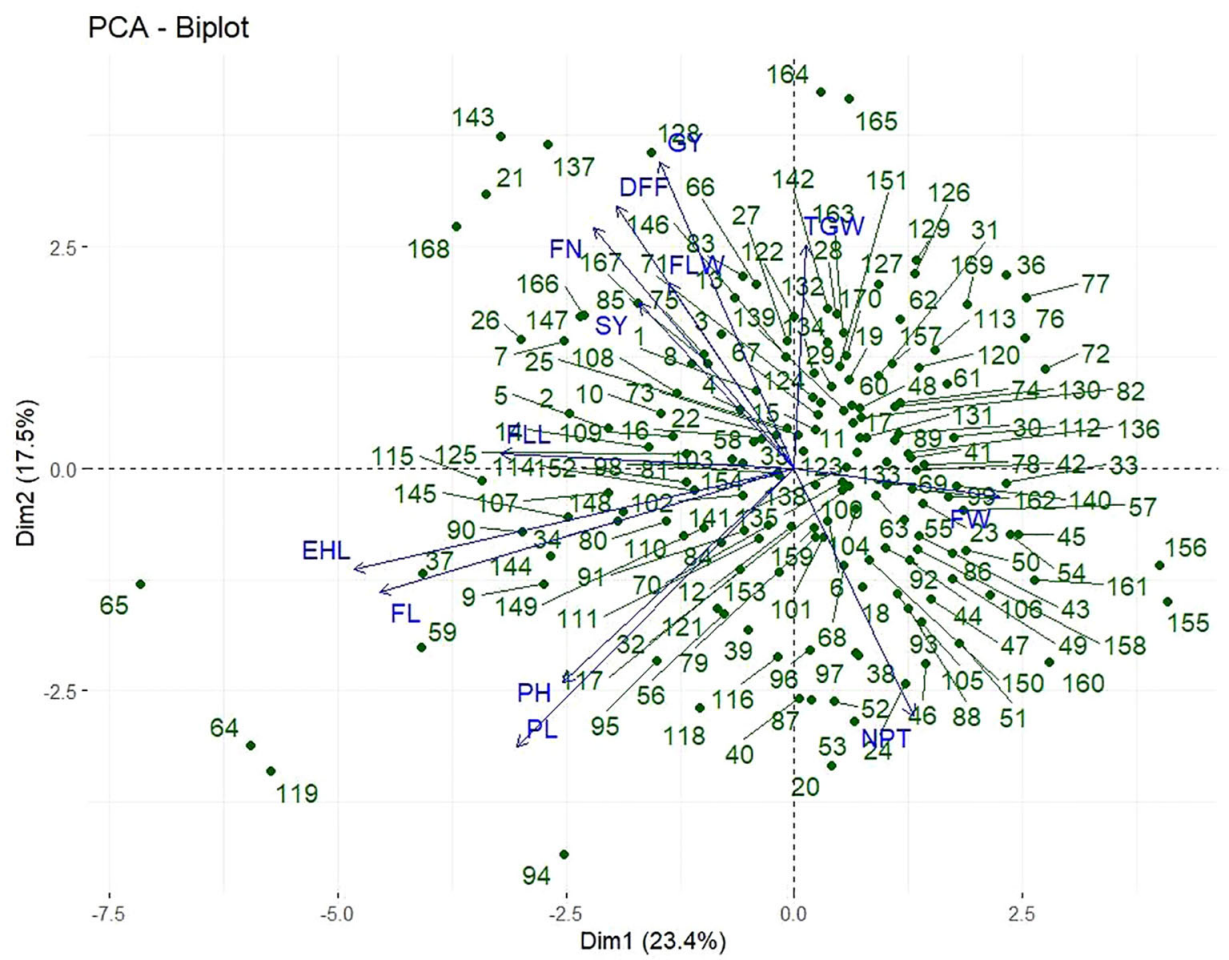
PCA biplot of first two components of 170 finger millet genotypes based on 13 quantitative traits.

PCA aids in identifying key components that contribute to phenotypic diversity and supports trait-based targeted breeding programs. The current investigation of 170 finger millet genotypes utilized PCA, which identified five components with eigenvalues exceeding 1, accounting for 70.1% of the total variation. The initial three components, PC1, PC2, and PC3, accounted for more than half (52.5%) of the phenotypic variation. Ketema et al. (2025) reported analogous findings, indicating that three components accounted for 60.5% of the variation. Sharma et al. (2022) and Ladumor et al. (2021) reported significant contributions of the first principal components, underscoring their importance in identifying distinguishing traits that account for diversity. This research identifies finger number, finger length, number of productive tillers, and grain yield as significant contributors to variation across the principal components, suggesting their potential utility in selection indices. The PCA biplot (**Figure 8**) demonstrated a strong correlation among days to 50% flowering, finger number, flag leaf width, grain yield, and stover yield, particularly with genotypes IC0053724, IC0087526, IC0473148, IC0049976, PRM2, IC0477739, IC0475312, and IC0473404 positioned along these vectors. There was a positive correlation between finger length and head ear length, with genotypes IC0340180, IC0473381, IC0340127, IC0474803, IC0474809, IC0473373, IC0041819, and IC0474741 linked to this relationship. Plant height and peduncle length formed a distinct group exhibiting a strong correlation and association with genotypes IC0474560 and IC0473327. Nagaraja et al. (2023) reported comparable genotype contributions through PCA, indicating that GE 5951 and GE 5957 exhibited positive contributions to PC1 and PC2, whereas GE 5949 and GE 4764 demonstrated genetic differentiation, suggesting their potential utility in hybridization. The results of variance analysis, diversity indices, correlation studies, cluster classification, and PCA indicate significant phenotypic and genetic diversity among the evaluated finger millet genotypes. The findings are crucial for guiding parental selection, prioritizing traits, and developing breeding strategies aimed at enhancing genetic gain in finger millet.

The combined results of variance analysis, diversity indices, correlation research, cluster classification, and PCA demonstrate extant phenotypic and genetic diversity among the tested finger millet genotypes.

## Conclusion

Ample phenotypic variations were observed among the genotypes for the 27 DUS descriptors of finger millet. This study’s results will enhance the descriptor system and refine the DUS test guidelines for finger millet by identifying new reference genotypes for essential DUS descriptors. This will serve as a reference for the further utilization of finger millet germplasm resources and the genetic enhancement of economic traits, thereby strengthening the theoretical foundation for breeding new varieties of finger millet. The identified traits have significant potential to enhance cultivar improvement and germplasm conservation, thereby contributing to the increased productivity of finger millet. This study focused on phenotypic evaluation, although informative and cost-effective for initial screening may not adequately represent the underlying genetic variation. Incorporating genotypic data would enhance the associations between traits and genotypes, thereby increasing selection accuracy and facilitating the identification of parental lines for breeding programs. The phenotypic variation observed in the finger millet diversity panel may be employed in genome-wide association studies to identify significant marker-trait associations (MTAs) for key DUS traits.

## Data Availability

The original contributions presented in the study are included in the article/**Supplementary Material**. Further inquiries can be directed to the corresponding author.
