# Supplementary material for: Quantifying genetic variation for DUS descriptors in diverse finger millet germplasm evaluated under semi-arid Bundelkhand region of India
Source: Front Plant Sci. 2026 Feb 11;17:1704652. doi: 10.3389/fpls.2026.1704652 (PMC12932421; doi:10.3389/fpls.2026.1704652)
Supplement: Supplementary file 1 [file Table1.docx]

Supplementary table 1: List of various genotypes and their biological status used in the current investigation

| **S. No** | **Genotype Name** | **Origin** | **Biological Status** | **S. No** | **Genotype Name** | **Origin** | **Biological Status** |
| --- | --- | --- | --- | --- | --- | --- | --- |
| 1 | IC0045878 | Tamil Nadu | Unknown | 86 | IC0473151 | Andhra Pradesh | Landrace |
| 2 | IC0049940 | Karnataka | Unknown | 87 | IC0474601 | Unknown | Unknown |
| 3 | IC0049976 | Karnataka | Unknown | 88 | IC0473165 | Andhra Pradesh | Landrace |
| 4 | IC0050000 | Karnataka | Unknown | 89 | IC0473382 | Tamil Nadu | Landrace |
| 5 | IC0065595 | Unknown | Landrace | 90 | IC0473381 | Tamil Nadu | Landrace |
| 6 | IC0065632 | Tamil Nadu | Landrace | 91 | IC0473335 | Assam | Landrace |
| 7 | IC0065998 | Unknown | Unknown | 92 | IC0477486 | Unknown | Unknown |
| 8 | IC0066235 | Unknown | Unknown | 93 | IC0041815 | Madhya Pradesh | Unknown |
| 9 | IC0066588 | Unknown | Unknown | 94 | IC0053735 | Chhattisgarh | Unknown |
| 10 | IC0073581 | Unknown | Unknown | 95 | IC0477471 | India | Landrace |
| 11 | IC0087463 | Tamil Nadu | Unknown | 96 | IC0473337 | Assam | Landrace |
| 12 | IC0087464 | Tamil Nadu | Unknown | 97 | IC0473327 | Uttar Pradesh | Landrace |
| 13 | IC0087480 | Unknown | Unknown | 98 | IC0474700 | Unknown | Unknown |
| 14 | IC0087502 | Himachal Pradesh | Unknown | 99 | IC0474548 | Unknown | Unknown |
| 15 | IC0087526 | Andhra Pradesh | Elite Line | 100 | IC0473320 | Uttar Pradesh | Landrace |
| 16 | IC0087549 | Karnataka | Landrace | 101 | IC0473321 | Uttar Pradesh | Landrace |
| 17 | IC0203961 | Unknown | Unknown | 102 | IC0474736 | Unknown | Unknown |
| 18 | IC0203975 | Unknown | Unknown | 103 | IC0473183 | Bihar | Landrace |
| 19 | IC0204143 | Unknown | Unknown | 104 | IC0473193 | Bihar | Landrace |
| 20 | IC0204144 | Unknown | Unknown | 105 | IC0474527 | Unknown | Unknown |
| 21 | IC0204147 | Unknown | Unknown | 106 | IC0473398 | Tamil Nadu | Landrace |
| 22 | IC0206159 | Unknown | Unknown | 107 | IC0473376 | Tamil Nadu | Landrace |
| 23 | IC0206162 | Unknown | Unknown | 108 | IC0473388 | Tamil Nadu | Landrace |
| 24 | IC0206164 | Unknown | Unknown | 109 | IC0473387 | Tamil Nadu | Unknown |
| 25 | IC0206176 | Unknown | Unknown | 110 | IC0473386 | Tamil Nadu | Landrace |
| 26 | IC0206178 | Unknown | Unknown | 111 | IC0473168 | West Bengal | Landrace |
| 27 | IC0206180 | Unknown | Unknown | 112 | IC0477451 | Unknown | Unknown |
| 28 | IC0206184 | Unknown | Unknown | 113 | IC0477542 | Unknown | Unknown |
| 29 | IC0206191 | Unknown | Unknown | 114 | IC0474741 | Unknown | Unknown |
| 30 | IC0206201 | Unknown | Unknown | 115 | IC0474800 | Unknown | Unknown |
| 31 | IC0206209 | Unknown | Unknown | 116 | IC0479120 | Unknown | Unknown |
| 32 | IC0206211 | Unknown | Unknown | 117 | IC0479134 | Unknown | Unknown |
| 33 | IC0206212 | Unknown | Unknown | 118 | IC0479168 | Unknown | Unknown |
| 34 | IC0340127 | Maharashtra | Unknown | 119 | IC0473405 | Tamil Nadu | Landrace |
| 35 | IC0340136 | Unknown | Unknown | 120 | IC0479016 | Unknown | Unknown |
| 36 | IC0340138 | Unknown | Unknown | 121 | IC0473403 | Tamil Nadu | Landrace |
| 37 | IC0340180 | Unknown | Unknown | 122 | IC0474599 | Unknown | Unknown |
| 38 | IC0476076 | Unknown | Unknown | 123 | IC0474810 | Unknown | Unknown |
| 39 | IC0476710 | Unknown | Unknown | 124 | IC0477597 | Unknown | Unknown |
| 40 | IC0476818 | Unknown | Unknown | 125 | IC0474963 | Unknown | Unknown |
| 41 | IC0476359 | Unknown | Unknown | 126 | IC0478962 | Unknown | Unknown |
| 42 | IC0475654 | Unknown | Unknown | 127 | IC0041805 | Maharashtra | Unknown |
| 43 | IC0476242 | Unknown | Unknown | 128 | IC0587935 | Karnataka | Landrace |
| 44 | IC0476315 | Unknown | Unknown | 129 | IC0477739 | Unknown | Unknown |
| 45 | IC0476418 | Unknown | Unknown | 130 | IC0053730 | Chhattisgarh | Unknown |
| 46 | IC0476006 | Unknown | Unknown | 131 | IC0474547 | Unknown | Unknown |
| 47 | IC0475632 | Unknown | Unknown | 132 | IC0477559 | Unknown | Unknown |
| 48 | IC0475697 | Unknown | Unknown | 133 | IC0477547 | Unknown | Unknown |
| 49 | IC0475978 | Unknown | Unknown | 134 | IC0477546 | Unknown | Unknown |
| 50 | IC0475457 | Unknown | Unknown | 135 | IC0479004 | Unknown | Unknown |
| 51 | IC0475053 | Unknown | Unknown | 136 | IC0478978 | Unknown | Unknown |
| 52 | IC0475334 | Unknown | Unknown | 137 | IC0479078 | Unknown | Unknown |
| 53 | IC0474887 | Unknown | Unknown | 138 | IC0041796 | Maharashtra | Unknown |
| 54 | IC0478790 | Unknown | Unknown | 139 | IC0041808 | Maharashtra | Unknown |
| 55 | IC0283409 | Elite line | Bihar | 140 | IC0053724 | Chhattisgarh | Unknown |
| 56 | IC0477845 | Unknown | Unknown | 141 | IC0053716 | Chhattisgarh | Unknown |
| 57 | IC0477868 | Unknown | Unknown | 142 | IC0474709 | Unknown | Unknown |
| 58 | IC0477899 | Unknown | Unknown | 143 | IC0474531 | Unknown | Unknown |
| 59 | IC0477322 | Unknown | Unknown | 144 | IC0474797 | Unknown | Unknown |
| 60 | IC0477424 | Unknown | Unknown | 145 | IC0473386 | Tamil Nadu | Landrace |
| 61 | IC0477445 | Unknown | Unknown | 146 | IC0041819 | Chhattisgarh | Unknown |
| 62 | IC0477450 | Unknown | Unknown | 147 | IC0473404 | Tamil Nadu | Landrace |
| 63 | IC0041810 | Maharashtra | Unknown | 148 | IC0473375 | Tamil Nadu | Landrace |
| 64 | IC0473384 | Tamil Nadu | Landrace | 149 | IC0473373 | Tamil Nadu | Landrace |
| 65 | IC0477708 | Unknown | Unknown | 150 | IC0473369 | Tamil Nadu | Landrace |
| 66 | IC0477572 | Unknown | Unknown | 151 | IC0473367 | Andhra Pradesh | Landrace |
| 67 | IC0474557 | Unknown | Unknown | 152 | IC0473357 | Madhya Pradesh | Landrace |
| 68 | IC0474564 | Unknown | Unknown | 153 | IC0473358 | Sikkim | Landrace |
| 69 | IC0474907 | Unknown | Unknown | 154 | IC0473359 | Sikkim | Landrace |
| 70 | IC0473364 | Andhra Pradesh | Elite Line | 155 | IC0283451 | Bihar | Elite line |
| 71 | IC0473365 | Andhra Pradesh | Elite Line | 156 | IC0283454 | Bihar | Elite line |
| 72 | IC0478977 | Unknown | Unknown | 157 | IC0298448 | Orissa | Unknown |
| 73 | IC0479039 | Unknown | Unknown | 158 | IC0298482 | Orissa | Unknown |
| 74 | IC0479049 | Unknown | Unknown | 159 | IC0346263 | Bihar | Elite line |
| 75 | IC0478946 | Unknown | Unknown | 160 | IC0347251 | Bihar | Elite line |
| 76 | IC0475048 | Unknown | Unknown | 161 | IC0347252 | Bihar | Elite line |
| 77 | IC0475049 | Unknown | Unknown | 162 | IC0347254 | Bihar | Elite line |
| 78 | IC0475098 | Unknown | Unknown | C1 | GPU-28 | Improved cultivar | Karnataka |
| 79 | IC0475041 | Unknown | Unknown | C2 | GPU-66 | Improved cultivar | Karnataka |
| 80 | IC0474809 | Unknown | Unknown | C3 | GPU-67 | Improved cultivar | Karnataka |
| 81 | IC0474803 | Unknown | Unknown | C4 | MR-1 | Improved cultivar | Karnataka |
| 82 | IC0474959 | Unknown | Unknown | C5 | MR-6 | Improved cultivar | Karnataka |
| 83 | IC0475312 | Unknown | Unknown | C6 | ML-365 | Improved cultivar | Karnataka |
| 84 | IC0474560 | Unknown | Unknown | C7 | PRM-2 | Improved cultivar | Uttarakhand |
| 85 | IC0473148 | Andhra Pradesh | Landrace | C8 | VR-929 | Improved cultivar | Andhra Pradesh |
